# Patient Safety Culture and Safety Attitudes in the Estonian Context: Simultaneous Bilingual Cultural Adaptation and Validation of Instruments

**DOI:** 10.3389/ijph.2024.1607392

**Published:** 2024-09-04

**Authors:** Signe Asi, Hiske Calsbeek, Mari Katariina Kangasniemi, Mare Vähi, Kaja Põlluste

**Affiliations:** ^1^ Institute of Clinical Medicine, University of Tartu, Tartu, Estonia; ^2^ Department of IQ Healthcare, Radboud University Medical Centre, Nijmegen, Netherlands; ^3^ Department of Nursing Science, Faculty of Medicine, University of Turku, Turku, Finland; ^4^ Institute of Mathematical Statistics, University of Tartu, Tartu, Estonia

**Keywords:** attitude, healthcare surveys, organizational culture, patient safety, validation study

## Abstract

**Objectives:**

This study aimed to simultaneously and bilingually validate the Hospital Survey on Patient Safety Culture (HSOPSC 2.0) and the Safety Attitudes Questionnaire (SAQ).

**Methods:**

The validation included translation, cultural adaptation, and assessment of validity and consistency. Data were collected in three hospitals in 2022 via online and paper surveys, with Estonian- and Russian-speaking employees participating.

**Results:**

In total, 579 (30%) participants from the three hospitals completed both questionnaires. Among them, 293 (51%) were Russian-speaking and 286 (49%) were Estonian-speaking. Cronbach’s αhy for HSOPSC 2.0 was ≥0.60, except in the Russian version for the three dimensions. Cronbach’s α for SAQ was ≥0.60, except in the Russian version for one dimension. Pearson’s correlations of the Estonian HSOPSC 2.0 ranged from 0.26 to 0.60 and in the Russian version from 0.18 to 0.47.

**Conclusion:**

The validity of the HSOPSC 2.0 and SAQ questionnaires was confirmed in the Estonian versions. Minor corrections were recommended for the Russian. Both versions are considered suitable for assessing PSC in Estonian hospitals.

## Introduction

Patient safety culture (PSC) in hospitals is fundamental to ensuring patient wellbeing, improving the quality of care, and engaging healthcare professionals in fostering an environment conducive to continuous improvement [1–3]. PSC is defined as an individual and organizational behavior pattern based on shared beliefs, attitudes, and values aimed at consistently minimizing patient harm [4]. PSC assessments are crucial for identifying areas of improvement and ensuring safe treatment for hospital patients. Implementing safety attitudes surveys enables organizations to proactively assess employees’ perceptions of safety culture. Addressing identified concerns promptly demonstrates a commitment to fostering a positive safety culture, thereby gaining employee buy-in and support for safety initiatives [1, 2, 5].

Various tools have been developed to assess PSC and attitudes [2]. The Hospital Survey on Patient Safety Culture (HSOPSC) and the Safety Attitudes Questionnaire (SAQ) capture aspects of safety culture within healthcare settings, such as communication openness, teamwork, and leadership support for safety initiatives. The HSOPSC, developed in 2004, has been validated in over 95 countries in different clinical contexts. The SAQ was developed in 2006 and a short version of SAQ is accessible, quick to complete, and available in multiple languages. Both questionnaires stand out as the most widely used and evaluated tools for measuring safety culture in healthcare comparisons and enable the monitoring of changes over time [4, 6].

In Estonia, patient safety in hospitals has been guided by two documents: the Patient Safety Research and Development Strategy (2022–2026) and the Health Development Plan (2020–2030). Prioritizing PSC activities in healthcare, as well as emphasizing their importance, are crucial steps to enhance the quality of healthcare systems [7, 8]. However, there has been a lack of opportunities to assess PSC in the absence of validated measurement instruments. By introducing validated tools such as the HSOPSC 2.0 and SAQ, it becomes possible to evaluate the PSC in Estonian hospitals from the perspective of employees. This assessment is needed to identify organizational weaknesses, plan systemic changes to promote a positive PSC, and contribute to improving patient safety in Estonian hospitals [1, 5, 7].

The use of two different validated instruments broadens the ability to measure various dimensions of PSC comprehensively and enables in-depth exploration of the phenomenon under investigation [2, 9]. Despite Estonian being Estonia’s official language, the country also has a significant number of employees who prefer Russian. It was thus considered necessary to validate the instruments in both languages. Therefore, the research aimed to simultaneously and bilingually culturally adapt and validate the HSOPSC 2.0 and SAQ questionnaires in the Estonian context.

## Methods

The study consisted of three phases: questionnaire translation, adaptation, and validation. The first phase included the initial review of the instruments (face validity), followed by the translation of the instruments from the original English version into Estonian and Russian. The second phase involved cultural adaptation, where the clarity and relevance of each questionnaire were assessed, also known as content validity. In the third phase, cross-sectional data were collected to evaluate the internal consistency and construct validity, including the structural and convergent validity of the instruments [10–13]. The methodological quality of the study was assessed using the adapted COSMIN checklist [14]. The study was approved by the Research Ethics Committee of the University of Tartu (decision 347/T-3).

### Instruments in This Study

**The Hospital Survey on Patient Safety Culture (HSOPSC)** version 1.0, compiled by the Agency for Healthcare Research and Quality (AHRQ) in 2004, has a revised version, the HSOPSC 2.0 (2019). The aim of the HSOPSC 2.0 is to measure the current state of the organization’s safety culture, identify strengths and areas in need of development in safety culture, and thereby increase employee awareness of patient safety. The HSOPSC 2.0 questionnaire consists of 32 items divided into 10 different subscales and has been reported to have good internal consistency and psychometric properties. Item responses were measured with 5-point agreement scales ranging from 1 = strongly disagree to 5 = strongly agree, or frequency from 1 = never to 5 = always. Written consent was obtained for use of the HSOPSC 2.0 instrument [4, 10, 15].

**The Safety Attitudes Questionnaire (SAQ) short-form** was developed by Sexton et al. to measure organizational culture factors that influence how employees manage situations involving a risk of defective or erroneous action: safety climate, teamwork climate, working conditions, job satisfaction, stress recognition, and perceptions of safety management [6]. The full version comprises 60 items and the short version contains 30 core items, with four of them answered separately for both hospital and unit levels, resulting in a total of 36 items. The questionnaire applies a 5-point Likert-type scale that ranges from 1 = disagree strongly to 5 = agree strongly for all items and is freely available [16–18].

#### Phase I: Face Validity and Translations of Instruments

**FIGURE 1:**
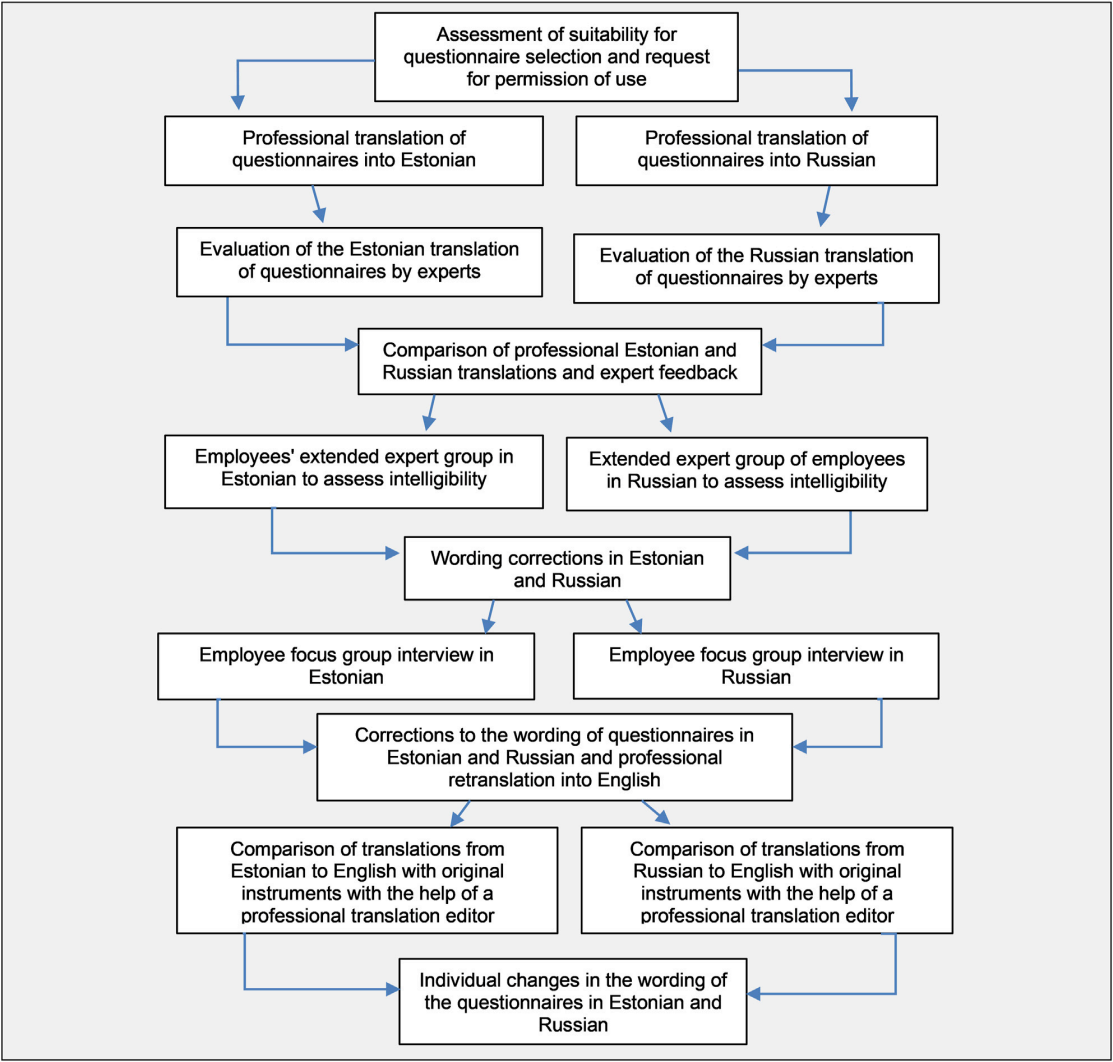
Face validity, translations, cultural adaptation, and content validity of the Hospital Survey on Patient Safety Culture 2.0 and Safety Attitudes Questionnaire (Simultaneous bilingual cultural adaptation and validation of patient safety culture instruments, Estonia, 2021–2022).

In the first phase, the research team evaluated that the selected instruments were suitable for cultural adaptation and validation in the Estonian healthcare context [12, 15]. Afterward, a professional translation company translated instruments from English to Estonian and Russian. Translated versions were then evaluated by experts: the Estonian versions were evaluated with three experts proficient in English, including a nursing director, medical director, and two physiotherapists. The Russian versions were assessed by two native Russian speakers, a nursing director, and a medical doctor/university teacher. The research team reviewed and adjusted translated questionnaires according to the expert feedback (Figure 1).

#### Phase II: Cultural Adaptation and Content Validity of Instruments

The content validity of the instruments was evaluated by an extended expert group, translation editors, and focus group interviews including speakers of both Estonian and Russian. First, an extended expert group was convened to assess the clarity of the questions and provide recommendations for any necessary wording adjustments. The two expert groups in Estonian (n = 8) and Russian (n = 8) consisted of representatives from the target audience, working in the three involved hospitals. In the Estonian-speaking extended expert group (n = 8), the members included: one physician, one midwife, three healthcare support specialists, one nursing assistant, one administrative worker, and one cleaning service worker. The Russian-speaking expert group (n = 8) comprised: two physicians, one nurse, three nursing assistants, one healthcare support specialist, and one administrative worker. The phrasing of instruments was revised based on the feedback from the extended expert group.

Second, the focus group interviews were carried out to evaluate cultural relevance and linguistic appropriateness. Participants provided feedback on the clarity, understandability, and appropriateness of the language and cultural references used in the items. In the Russian-speaking focus group interview, six employees participated: two nursing assistants, two nurses, a nurse manager, and a social worker. The Estonian-speaking focus group consisted of a social worker, a nurse, a nursing assistant, and an administrative worker. Interviews were carried out online because of the COVID-19 pandemic. Based on feedback from the focus group interviews, four corrections on phrasing were made to the Estonian version, and five corrections were made to the Russian version.

Finally, the instruments were back-translated into English by a translation agency. A comparison between the original instruments and the Estonian and Russian versions involved two professional language editors. After final adjustments and sentence corrections, the cultural adaptation of the instruments was completed (Figure 1).

#### Phase III: Internal Consistency and Construct Validity of Instruments

##### Data Collection

Internal consistency and construct validity were tested in a cross-sectional study assessment. Data was collected in three hospitals in the year 2022. During the data collection period, there were altogether 1,948 employees working in the hospitals, including healthcare staff such as physicians, nurses, nursing assistants, radiologists, laboratory technicians, and physiotherapists. In addition, there were also social workers, cleaning personnel, transportation and administrative staff, and middle-level managers.

Data were collected in collaboration with hospital contact persons using electronic and paper instruments. The hospital contact person shared an electronic link of instruments for employees, using the REDCap program. Paper questionnaires (altogether 1,200, including 635 in Estonian and 565 in Russian) were delivered to the departments by the contact person and participants were asked to put filled questionnaires in sealed envelopes placed in designated boxes within departments. Data collection was conducted in two periods, first in April 2022 and again from November to December 2022. Data were collected using the HSOPSC 2.0 and SAQ questionnaires and supplemented with background information, including personal professional background and workplace characteristics.

##### Data Analysis

The data were analyzed by descriptive and advanced statistics and performed using the SPSS 29.0 statistical software for Windows. Background variables were analyzed by descriptive methods using percentages for frequency distributions and associations were found between background data and the gap in respondents’ scores. For analysis, responses to negatively worded questions were reversed. High safety culture levels were “Agree” or “Strongly Agree” in HSOPSC and “Agree Slightly” or “Agree Strongly” in SAQ. Lower levels were indicated by “Strongly Disagree” or “Disagree” in HSOPSC and “Disagree Strongly” or “Disagree Slightly” in SAQ [4, 6, 12, 19].

Internal consistency, determined by Cronbach’s α coefficient, targeted values of ≥0.6, which are considered acceptable. Construct validity of the instruments was assessed to ensure they accurately measure the intended concepts, focusing on both structural and convergent validity measures. Structural validity was evaluated using confirmatory factor analysis (CFA) conducted with SAS 9.4. Convergent validity was assessed using the Pearson correlation coefficient. Mean scores, standard deviation (SD), and response rates were calculated based on the received questionnaires [20, 21].

## Results

In total, there were 579 (30%) respondents from the three hospitals, of which 293 (51%) completed the questionnaire in Russian and 286 (49%) in Estonian. For the HSOPSC 2.0, there were 293 in Russian languages and 286 respondents in Estonian, while for the SAQ, 241 respondents completed it in Russian, and 227 in Estonian (Table 1).

**TABLE 1:** Participants’ background characteristics (Simultaneous bilingual cultural adaptation and validation of patient safety culture instruments, Estonia, 2021–2022).

	Characteristics of the responders	n (%)
Profession positions	Nurses and midwives Nursing assistants Healthcare support specialists (physiotherapist, radiology technician, etc.) Support and administrative staff (quality service, financial service, office, etc.) Physicians Other positions (security, transport, etc.) Interns and volunteers Missing information	205 (36%) 109 (19%) 71 (12%) 69 (12%) 54 (9%) 46 (8%) 1 (0%) 24 (4%)
Working unit	Psychiatry Rehabilitation Support and administrative services (quality, financial service, office, etc.) Department of internal medicine Non-medical support services (security, transport) Outpatient care unit Medical-support services (laboratory, radiology) Working in multiple departments Women’s health and maternity Nursing care Vaccination and infection control center Intensive care unit Pediatrics Missing information	90 (16%) 89 (15%) 29 (5%) 26 (4%) 21 (4%) 20 (3%) 17 (3%) 12 (2%) 9 (2%) 7 (1%) 3 (1%) 2 (0%) 1 (0%) 253 (44%)
Leading position	In a non-leading position In a leading position Missing information	458 (79%) 79 (14%) 42 (7%)
Number of years in hospital	Less than a year 1–5 years 6–10 years 11 or more years Missing information	59 (10%) 137 (24%) 86 (15%) 262 (45%) 35 (6%)
Number of years in department	Less than a year 1–5 years 6–10 years 11 or more years Did not respond	83 (10%) 157 (24%) 92 (15%) 212 (45%) 35 (6%)
Working hours per week	Full-time (40 h per week) More than full-time Part-time (less than 40 h per week) Full time plus additional employment elsewhere Missing information	334 (57%) 109 (19%) 79 (14%) 21 (4%) 36 (6%)
Direct communication with the patient	Direct communication with the patient No direct communication with the patient Missing information	431 (75%) 111 (19%) 37 (6%)
Concerning age	Patients of different ages Adults Elderly Children No contact with the patients Missing information	259 (45%) 102 (18%) 63 (11%) 21 (4%) 69 (12%) 65 (10%)

### Inadequately Responded Items

The highest non-response rates were observed in the HSOPSC 2.0-EST instrument for questions D1 (44%) “When a mistake is caught and corrected before reaching the patient, how often is this reported?,” D2 (42%) “When a mistake reaches the patient and could have harmed the patient, but did not, how often is this reported?,” and C5 (39%) “When staff in this unit see someone with more authority doing something unsafe for patients, they speak up,” and in the Russian version same items for C5 (29%), D2 (26%), and D1 (25%). In the SAQ-EST, the highest non-response rates were for questions saq34 (59%) “I experience good collaboration with staff physicians in this clinical area,” saq8 (29%) “Medical errors are handled appropriately in this clinical area,” and saq31 (29%) “Problem personnel are dealt with constructively by our: unit management/hospital management,” and in the Russian version for saq34 (44%) “I experience good collaboration with staff physicians in this clinical area,” saq31 (24%) “All the necessary information for diagnostic and therapeutic decisions is routinely available to me,” and saq2 (23%) “In this clinical area, it is difficult to speak up if I perceive a problem with patient care.”

### Internal Consistency of Instruments

The Cronbach’s α values (Table 2) were 0.60 or higher for all HSOPSC 2.0 dimensions, except for the Russian-language version (HSOPSC 2.0-RUS) dimensions Teamwork (α = 0.42), Staffing and Work Pace dimension (α = 0.27), and Organizational Learning—Continuous Improvement (α = 0.59). Similarly, Cronbach’s α values for the SAQ were ≥0.60, except for the Safety Climate dimension in the Russian-language version (SAQ-RUS) (α = 0.57).

**TABLE 2:** Cronbach’s α coefficient of the Hospital Survey on Patient Safety Culture 2.0 and the Safety Attitudes Questionnaire in Estonian and Russian compared to the original version [4, 6] and previous studies [10, 11, 15–18, 21] (Simultaneous bilingual cultural adaptation and validation of patient safety culture instruments, Estonia, 2021–2022).

	Estonian version	Russian version	Original version	Previous studies
**HSOPSC 2.0 dimensions**				
Teamwork	0.63	0.42	0.76	0.68–0.77
Staffing and work pace	0.67	0.27	0.67	0.47–0.74
Organizational learning — continuous improvement	0.72	0.59	0.76	0.60–0.76
Response to error	0.78	0.70	0.83	0.68–0.81
Supervisor, manager, or clinical leader support for patient safety	0.71	0.67	0.77	0.71–0.77
Communication about error	0.86	0.80	0.89	0.73–0.87
Communication openness	0.76	0.78	0.83	0.67–0.82
Reporting patient safety event	0.76	0.81	0.75	0.73–0.81
Hospital management support for patient safety	0.83	0.77	0.77	0.62–0.76
Handoffs and information exchange	0.67	0.68	0.72	0.50–0.76
**SAQ dimensions**				
Teamwork climate	0.83	0.77	0.70	0.69–0.76
Safety climate	0.65	0.57	0.73	0.76–0.87
Job satisfaction	0.83	0.89	0.86	0.84–0.87
Stress recognition	0.89	0.85	0.82	0.78–0.86
Perceptions of management	0.88	0.90	0.88	0.86–0.93
Working conditions	0.62	0.69	0.71	0.72–0.80

### Construct Validity of Instruments

To study the construct validity, we calculated the mean scores per dimension. The mean scores of the two instruments ranged from 3.56 to 4.42 for the SAQ and from 3.21 to 4.17 for the HSOPSC 2.0 (Table 3).

**TABLE 3:** Mean scores, standard deviation, and response rates for the domains of the Hospital Survey on Patient Safety Culture 2.0 and the Safety Attitudes Questionnaire (Simultaneous bilingual cultural adaptation and validation of patient safety culture instruments, Estonia, 2021–2022).

	Respondents (n)		Mean		SD	
	Estonia	Russia	Estonia	Russia	Estonia	Russia
**HSOPSC 2.0 (score range 3.21–4.17)**						
Teamwork	261	258	4.03	3.60	0.65	0.62
Staffing and work pace	235	249	3.54	3.18	0.77	0.54
Organizational learning—continuous improvement	187	243	3.21	3.55	0.81	0.65
Response to error	191	252	3.37	3.41	0.81	0.68
Supervisor, manager, or clinical leader support for patient safety	225	258	3.82	3.73	0.71	0.62
Communication about error	209	248	3.83	4.17	1.02	0.88
Communication openness	155	183	3.89	3.87	0.80	0.86
Reporting patient safety event	145	207	3.57	4.06	1.11	1.02
Hospital management support for patient safety	178	249	3.55	3.73	0.84	0.68
Handoffs and information exchange	154	223	3.59	3.61	0.76	0.68
**SAQ (score range 3.56–4.42)**						
Teamwork climate	153	164	4.12	4.01	0.76	0.6
Safety climate	129	173	3.86	3.87	0.86	0.68
Job satisfaction	210	217	4.42	4.31	0.65	0.71
Stress recognition	157	189	4.15	3.56	0.96	0.98
Perceptions of management	106	150	3.81	3.89	0.77	0.64
Working conditions	140	165	3.76	3.61	0.79	0.71

Pearson correlation coefficients for the Estonian HSOPSC 2.0 (HSOPSC 2.0-EST) ranged from 0.26 to 0.60 (*p* < 0.05). The strongest correlation was in the Organizational Learning—Continuous Improvement dimension (r = 0.60), while the weakest correlation was found in Staffing and Work Pace (r = 0.26). In the HSOPSC 2.0-RUS, correlations ranged from 0.18 to 0.47. The highest correlation was also found in Organizational Learning—Continuous Improvement (r = 0.47), and the lowest was in the Staffing and Work Pace dimension (r = 0.18). In the SAQ-RUS instrument, the highest correlation was observed in the Perceptions of Management dimension (r = 0.40), while in the Estonian-language instrument, it was in the Teamwork Climate dimension (r = 0.12).

Furthermore, correlations were calculated between each dimension of the instrument and the question, “How would you rate your unit/department in terms of patient safety?” For all dimensions of HSOPSC 2.0, except for the Staffing and Work Pace dimension (r = 0.17), the relationship with the overall rating ranged from 0.3 to 0.6.

Based on correlations between the HSOPSC 2.0 and SAQ questionnaires (Table 4), statistically significant correlations were observed between Teamwork/Teamwork Climate in HSOPSC 2.0 and Safety climate/Organizational Learning—Continuous Improvement (r = 0.30), Hospital Management Support for Patient Safety/Perceptions of Management (r = 0.41), Communication Openness/Safety Climate (r = 0.42), Communication Openness/Perceptions of Management (r = 0.39), and Supervisor, Manager, or Clinical Leader Support for Patient Safety/Perceptions of Management (r = 0.37).

**TABLE 4:** Summary of confirmatory factor analysis results for the hospital survey on patient safety culture 2.0 and the safety attitudes questionnaire in Estonian and Russian (Simultaneous bilingual cultural adaptation and validation of patient safety culture instruments, Estonia, 2021–2022).

Index	Index threshold values	HSOPSC 2.0 (Estonian and Russian)	SAQ (Estonian and Russian)
/df		1.8	1.66
RMSEA (95% CI)	<0.08	0.0649 (0.0574–0.0723)	0.0585 (0.0507–0.0662)
SRMR	<0.08	0.0698	0.0620
GFI	>0.8	0.8038	0.8159

Correlations between similar dimensions of the two instruments ranged from 0.30 to 0.42. HSOPSC 2.0 dimensions such as Response to Error, Communication Openness, and Hospital Management Support for Patient Safety correlated with dimensions in the SAQ questionnaire such as Teamwork Climate, Safety Climate, and Perceptions of Management.

The construct validity is supported by factor analysis, and the goodness-of-fit indices show that the data fit the intended 10-factor model. In the confirmatory factor analysis (CFA) of HSOPSC (Table 4), χ2/df = 1.8, RMSEA = 0.06, and SRMR = 0.07. In the SAQ instrument, χ2/df = 1.66, RMSEA = 0.06, and SRMR = 0.06. RMSEA (Root Mean Square Error of Approximation) values of 0.06 for both HSOPSC and SAQ are below the recommended threshold of 0.08, indicating a good fit. SRMR (Standardized Root Mean Square Residual) reported values of 0.07 for HSOPSC and 0.06 for SAQ are below the threshold of 0.08.

## Discussion

This study validated the HSOPSC 2.0 and SAQ instruments in the Estonian healthcare context and assessed the psychometric properties of developed Estonian and Russian versions. Findings indicated strong internal consistency and validity, suggesting these instruments effectively capture employees’ perceptions of PSC in Estonian hospitals with diverse linguistic backgrounds.

### Translations, Cultural Adaptation, and Content Validity of Instruments

In this study, instruments were selected and simultaneously translated into two languages, a practice not documented in previous studies. While instruments have been concurrently validated before [9], this has not been done in different languages simultaneously, a method that proved effective in minimizing differences and enhancing reliability and validity. Based on the adapted COSMIN checklist [14], and drawing from other validation studies, all recommended stages of translation and cultural adaptation were carried out. The inclusion of diverse professional fields was crucial for assessing content validity, as varied perspectives significantly contribute to refining questions and increasing comprehensibility. Various specialists in Russian and Estonian, as well as experts and language editors, participated in the process, providing a strong assurance of the adequacy of the translations. The analysis of the collected research data indicated that most participants were nurses and midwives, followed by nursing assistants, healthcare support specialists, support and administrative staff, and physicians. This reflects the general staff composition, where nurses make up the largest proportion of hospital staff. In various countries, the samples of adapted and validated instruments differed both in size and profession. For instance, Suryani et al. [10], Filiz et al. [11], and Lee et al. [15] conducted a psychometric study exclusively among nurses, excluding the rest of hospital staff from the validation process.

As patient safety culture in Estonia is still nascent, this could have influenced inadequately answered items during cultural adaptation. To determine why certain questions in both the Estonian and Russian instruments were left unanswered, a qualitative study should be conducted. For instance, in the HSOPSC 2.0-EST and RUS, section D, where the questions were about reporting incidents, they were repeatedly rephrased based on the recommendations of the expert group and language editors, and important information was highlighted for better understanding, following a suggestion made during the extended focus group but had the highest non-response rate.

The SAQ-RUS and EST had the highest non-response rate for the item regarding collaboration with pharmacists, which suggests that collaboration with pharmacists in the department or unit is uncommon. Also, the items asking whether problems are dealt with constructively by unit management or hospital management had a high non-response rate, as well as an item indicating that management does not knowingly compromise patient safety in the hospital. This suggests that employees may not be very aware of the hospital management’s activities related to patient safety. The same problem appears in Skjeggestad et al. [18], where the highest percentage of missing items was related to perceptions of management. In European hospitals, the predominant hierarchical structure, particularly the top-down management model, may hinder unit staff from raising concerns or engaging in discussions with the management, as mentioned by Nguyen et al. [13].

### Internal Consistency of Instruments

Cronbach’s α coefficients demonstrated satisfactory internal consistency (equal to or greater than 0.60) across all dimensions of the HSOPSC 2.0. Excluding low Cronbach α results in the HSOPSC 2.0-RUS for Teamwork, Staffing and Work Pace, and Organizational Learning—Continuous Improvement dimensions, overall Cronbach α scores were considered satisfactory. Notably, discrepancies were observed in those dimensions, attributable to variations in the translation of the HSOPSC 2.0-RUS. Specifically, within the Estonian adaptation, the dimension of Teamwork in a statement, addressing the elongation of workdays and its impact on patient safety, diverged in content from its Russian counterpart “The staff works for longer hours to improve patient safety” while the original version is “Staff in this unit work longer hours than is best for patient care.” This dissimilarity was substantiated by a notably low Cronbach’s α value of 0.27 calculated for the Staffing and Work Pace dimension of the HSOPSC 2.0-RUS. In the same section, statement A5, examining the dependency on temporary, float, or PRN staff, remained unclear in both the Russian version focus and expert group interviews because, in the Estonian context, the prevalence of temporary staff in the Estonian healthcare system is uncommon. Interestingly, in the Estonian version, this item was not problematic. The same issue appears in Lee et al. where A5 was deleted because it does not fit with the national context and this item may seem confusing or irrelevant in Korean healthcare systems [15]. If questions A3 and A5 are excluded from the HSOPSC 2.0-RUS in the Staffing and Work Pace dimensions, the Cronbach’s alpha coefficient increases to 0.42. The Indonesian HSOPSC 2.0, validated by Suryani et al. [10] with factor loads ranging from 0.47 to 0.65, except for Communication Openness (α = 0.67) and Response to Error (α = 0.68). Lee et al. [15] designed the Korean version, removing an inapplicable item. Cronbach’s α values for nine composites ranged between 0.71 and 0.83, except for Staffing and Work Pace (α = 0.61). The Turkish HSOPSC 2.0 had Cronbach α values between 0.72 and 0.82 [11] and Brazilian 0.47–0.87 [21].

In the SAQ, Cronbach’s α values of the dimensions were ≥0.6, indicating satisfactory validity, except for the safety climate dimension in the SAQ-RUS. There is a slight translation difference in the item “I am encouraged by my colleagues to report any patient safety concerns I may have” In Estonian, it was translated as “Colleagues encourage me to report all patient safety-related issues,” and in Russian as “My colleagues encourage me to report any patient safety concerns that may arise for me.” This may seem broader in meaning in Estonian than in Russian, but data analysis did not reveal significant differences in responses to these items. In the SAQ-RUS Cronbach’s alpha values ranged 0.57–0.90. Additionally, excluding the low result in the Safety Climate dimension of the SAQ-RUS (α = 0.57), Cronbach’s alpha scores were satisfactory. In other studies, Cronbach alphas ranged from 0.73 to 0.87 [18], conducted by Skjeggestad et al., and in a study conducted in Denmark, the range was from 0.70 to 0.86 [16].

### Construct Validity of Instruments

The Pearson correlation coefficients for both the HSOPSC 2.0-EST and SAQ-EST indicated sufficient independence between the sub-scales and provided evidence of the validity of the instruments. Exceptionally high correlations were not observed. Correlations between similar dimensions of the two instruments ranged from 0.30 to 0.42, indicating a good correlation between the subscales as hypothesized. However, these correlations remained lower than expected in terms of statistically significant relationships. The dimensions of HSOPSC 2.0 were correlated with the SAQ dimensions. Similar results were identified in the study by De Carvalho et al. [9] where the Teamwork Climate (SAQ) domain was significantly correlated with five HSOPSC domains.

The construct validity, measured by structural and convergent validity, was confirmed after hypotheses testing. Construct validity was confirmed through factor analysis, and the fit indices indicated that the data matched the proposed 10-factor model. The summary of confirmatory factor analysis results for HSOPSC 2.0 and SAQ Estonian and Russian language questionnaires confirms a good fit and correlation between dimensions.

### Strengths and Limitations

The study had some limitations and strengths. The first limitation was the low participation rate, caused by fatigue among workers due to the COVID-19 pandemic. Therefore, additional data collection was conducted in the fall of 2022, which did not significantly increase the number of participants. Additionally, the low participation rate may also be associated with the absence of an option to save partially completed questionnaires in REDCap, or because filling out the questionnaires was too time-consuming. The second limitation was the novelty and the sensitivity of the topic, which was highlighted in both the focus group interviews and the extended expert group discussions. Additionally, if respondents do not consider the research topic important or do not understand the usefulness of the collected data, their motivation to participate may have been lower. This emphasizes the need for further training and clarification of patient safety issues for both medical and non-medical hospital staff.

The strength of the study was that the research team followed the adapted COSMIN guideline, ensuring strong methodological quality. Another strength was certainly the simultaneous validation of two patient safety culture instruments, which, on the one hand, was more complex but, on the other hand, allowing for the concurrent execution of data collection and analysis stages, resulting in significant time and resource savings, and enhancing reliability and validity.

## Implications for Clinical Practice and Further Research

In the Estonian context, two validated questionnaires for assessing PSC enable conducting a comprehensive national study to understand the current state of safety culture in Estonian hospitals. This study would involve collecting data from various hospitals across the country and comparing it with data from other countries. Additionally, if deficiencies in safety culture were identified through the safety culture study, effective intervention strategies could be developed and implemented.

### Conclusion

As a result of the validation process, there is evidence supporting the clarity, relevance, internal consistency, and construct validity of the Estonian and Russian versions of the HSOPSC 2.0 and SAQ questionnaires. These conclusions are based on data collected from medical and non-medical staff in three hospitals. Therefore, the validity of the HSOPSC 2.0 and SAQ questionnaires in Estonian was confirmed. However, minor adjustments were recommended for the Russian version, including the deletion or rewording of items A3 “Staff in this unit work longer hours than is best for patient care” and A5 “This unit relies too much on temporary, float, or PRN staff” from the HSOPSC 2.0 and saq12 “I am encouraged by my colleagues to report any patient safety concerns I may have” from the SAQ instrument. Both questionnaires are suitable for assessing patient safety culture from the perspective of hospital staff in Estonian hospitals and are available in both Estonian and Russian.
